# The lipid mediator lysophosphatidic acid induces folding of disordered peptides with basic amphipathic character into rare conformations

**DOI:** 10.1038/s41598-018-32786-4

**Published:** 2018-09-28

**Authors:** Tünde Juhász, Judith Mihály, Gergely Kohut, Csaba Németh, Károly Liliom, Tamás Beke-Somfai

**Affiliations:** 1 Institute of Materials and Environmental Chemistry, Research Centre for Natural Sciences, Hungarian Academy of Sciences, Magyar tudósok körútja 2., Budapest, H-1117 Hungary; 2 Department of Biophysics and Radiation Biology, Semmelweis University, Tűzoltó u. 37-47., Budapest, H-1094 Hungary

## Abstract

Membrane-active, basic amphipathic peptides represent a class of biomolecules with diverse functions. Sequentially close protein segments also show similar behaviour in several ways. Here we investigated the effect of the lipid mediator lysophosphatidic acid (LPA) on the conformation of structurally disordered peptides including extracellular antimicrobial peptides (AMPs), and calmodulin-binding motifs derived from cytosolic and membrane target proteins. The interaction with associated LPA resulted in gain of ordered secondary structure elements, which for most cases were previously uncharacteristic of the particular peptide. Results revealed mechanism of the LPA-peptide interactions with regulation of the lipid on peptide conformation and oligomerization in a concentration-dependent manner involving (1) relocation of tryptophan residues into the lipid cluster, (2) multiple contacts between the binding partners dictated by complex driving forces, (3) multiple peptide binding to LPA associates with an affinity in the low micromolar range, and (4) selectivity for LPA compared with structurally related lipids. In line with recent findings showing endogenous molecules inducing structural changes in AMPs, we propose that accumulation of LPA in signalling or pathological processes might modulate host-defense activity or trigger certain processes by direct interaction with cationic amphipathic peptide sequences.

## Introduction

Partially or fully unfolded peptide and protein sequences can be found in a diverse set of biological functions, and often contain a mix of cationic and apolar residues forming a basic amphipathic peptide. As one example, this type of sequence is characteristic also for antimicrobial peptides (AMPs), which most often exert their effects on membranes by disrupting their integrity *via* various, only partially understood mechanisms of action^1,2^. The positively charged residues may facilitate their binding to its place of action *i*.*e*. negatively charged microbial membrane surface *via* electrostatic attraction while the hydrophobic residues provide contact site to the apolar region within the lipid bilayer. AMPs, or host-defense peptides, as components of the innate immune system^3^, are present extracellularly and besides the above described bacterial membrane activity, may also function by targeting metabolic processes or intracellular components. Sharing similar structural propensities, well-characterized melittin and mastoparan, main components of bee and wasp venom, respectively, are also known for their antibacterial activity^4,5^. Closely related to these stand-alone peptides, the typical intracellular binding motif of key calcium sensor protein calmodulin (CaM) is also a peptide segment, sharing the basic amphipathic nature of the above AMPs. Calmodulin regulates the activity of a great number of targets including cytosolic and membrane proteins^6^, among them channels and pumps located in the plasma-membrane. The calmodulin-binding domain on target proteins is an at least partially disordered segment of ~25 residues with the ability to fold into a basic amphipathic helix upon binding to calmodulin^7^. Target peptide binding is oriented by the hydrophobic pockets on each of the two calmodulin domains as well as the nearby negatively charged protein residues while calmodulin itself provides a flexible platform for the interaction^8^. Due to fulfilling the not so specific requirements for the consensus sequences of a calmodulin-binding domain, melittin is extensively used to model calmodulin-target peptide interactions *in vitro*^9–12^.

AMP interaction with natural membranes is accompanied by peptide folding, and most often involves some kind of assembly, higher order oligomerization, of the peptide as well^1^. Besides natural membranes, lipids incorporated into model membranes like liposomes, lipid clusters, or micelles were also shown to force peptide folding^13^. Interaction with lipid species can easily drive conformational changes in polypeptides, proteins as well. Intrinsically disordered proteins were reported to undergo a disorder-to-order transition upon binding to a lipid partner^14^. In contrast, proteins with a well-defined, folded structure were often shown to partially unfold or even to lose their 3D structure in the presence of lipids, especially of micelle-forming ionic lipids^15^. The acidic detergent with a middle-long alkyl chain, sodium dodecyl sulphate (SDS), was widely used to mimic membrane environment, and to study structural determinants of peptide-lipid and protein-lipid interactions. However, SDS is a non-natural compound contrasted to the lipid mediator lysophosphatidic acid (LPA) present in living organisms. LPA shares several features with SDS involving a negatively charged head-group and a single hydrocarbon chain but contains a phosphoryl-group instead of the sulphate and a longer acyl chain. LPA is a signalling lipid with a plethora of physiological actions including response to injury, stimulation of cell migration, and proliferation in many cell types, whereas elevated LPA level in pathological conditions is associated with chronic inflammation, cancer, or atheroscelosis^16,17^. It can be formed both intra- and extracellularly upon the action of lipases, thus it is produced in the plasma-membrane in various signalling processes or in the blood by activated platelets. Its local accumulation induces the highest positive membrane curvature^18^, which can initiate membrane fission events *in vivo*, should be stabilized by partner molecules, and could be modelled by the micellar lipid state *in vitro*. The action of AMPs is also connected to generation of curvatures in membranes, facilitating local lipid phase separation, and resulting finally in disorganization/disintegration of the lipid bilayer^19^.

Herein we investigate the effect of LPA on the conformation of basic amphipathic peptides involving calmodulin binding domains of cytosolic and membrane target proteins as well as antimicrobial peptides. We demonstrate that associated LPA can effectively drive peptide folding to both helical and β-sheet structures with a preference for rather non-standard conformations. Structural variations caused by LPA and SDS are compared, and possible regulation of peptide function by the lipid mediator is discussed, too.

## Results and Discussion

### CD spectroscopic detection of LPA induced structural changes

**Figure 1:**
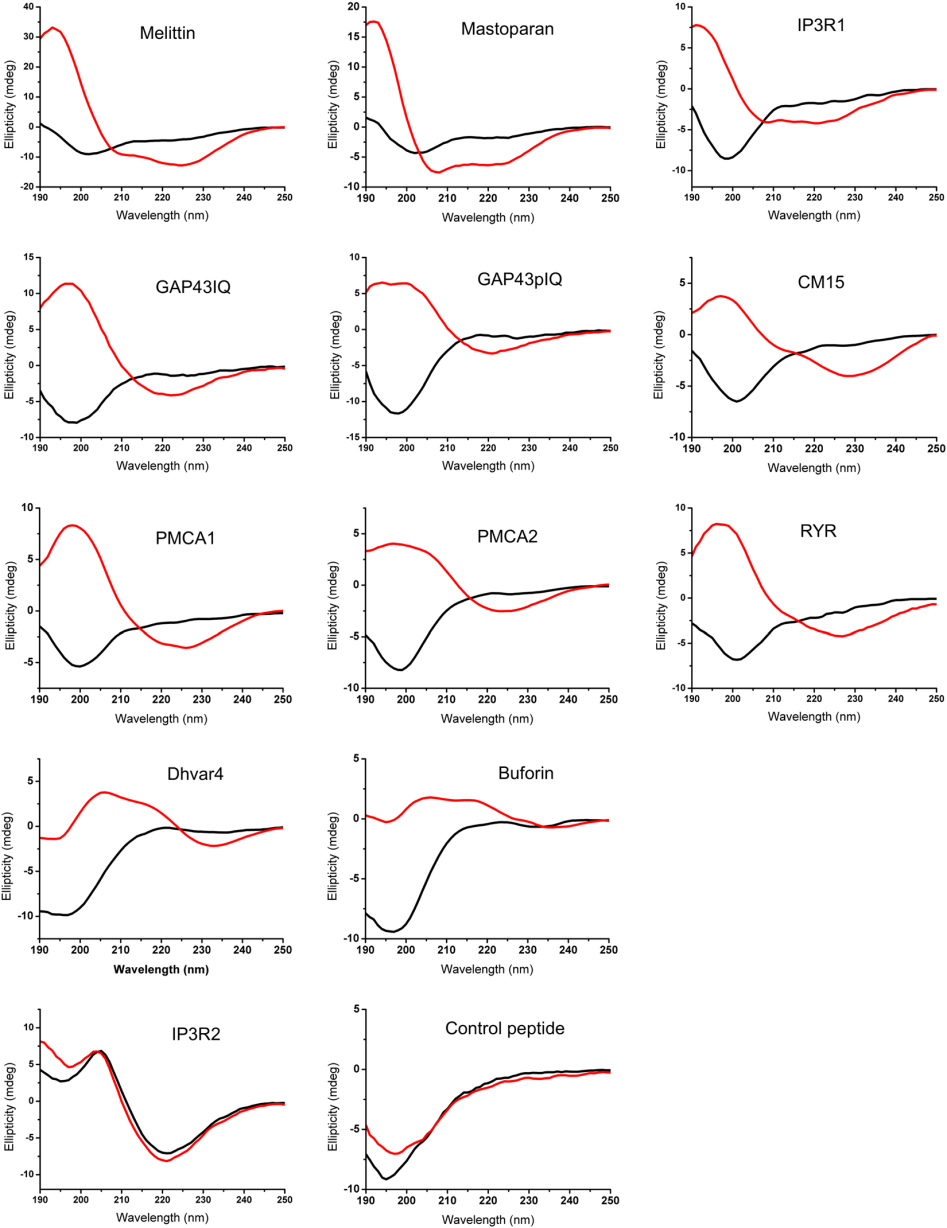
Far-UV CD spectra of the peptides in the absence (black) and presence (red) of LPA. Spectra were collected with and without 100 μM LPA under low-salt conditions. The induced secondary structure is mainly helical for melittin (25 µM), mastoparan (25 µM) and peptide IP3R1 (36 µM) (top row), rich in β-sheet for eight peptides (GAP43IQ (36 µM), GAP43pIQ (36 µM), CM15 (24 µM), PMCA1 (21 µM), PMCA2 (26 µM), RYR (34 µM), Dhvar4 (18 µM), buforin (24 µM); middle rows), while no remarkable change was detected for IP3R2 (36 µM) and the control peptide (36 µM) (bottom row). Note that ellipticity scales are different.

CD spectra recorded in the far-UV region provide useful data to assess the secondary structure of proteins and peptides. Spectra were collected for eleven peptides sharing a basic amphipathic character, and for a control peptide bearing several negatively charged residues (Table 1, Fig. 1). Most of the peptides showed a CD spectrum with a pronounced negative peak at around 200 nm, which is characteristic of a disordered structure, and were thus interpreted as unordered. It should be noted that secondary structure prediction for disordered peptides and proteins based on CD data can be ambiguous, since many prediction algorithms with different data sets containing mainly native globular proteins might fail to estimate the overall unordered structure correctly. The BeStSel analysis tool^20^ used here calculated approx. 30% antiparallel β-conformation with dominant right-twisted antiparallel sheet segments for disordered peptides with a main minimum below 200 nm. In contrast, peptides melittin, mastoparan, and CM15 showed a minimum at or slightly above 200 nm with a negative shoulder at 220 nm indicating some structural arrangement. The peptide IP3R2 displayed a strikingly different spectrum with a minimum at 220 nm, and a maximum at 200 nm, which indicated a folded structure with remarkable β-sheet content.

**Table 1:** Peptides used in the study.

Peptide	Type	Sequence	Residues	With LPA folding to
Melittin (MEL)	AMP	GIGAVL**K**VLTTGLPALISWI**KRK**RQQ-amide	26	α-helix
Mastoparan (MAS)	AMP	INL**K**ALAALA**KK**IL-amide	14	α-helix
CM15	AMP	**K**W**K**LF**KK**IGAVL**K**VL-amide	15	β-sheet
Dhvar4	AMP	**KR**LF**KK**LLFSL**RK**Y	14	β-sheet
Buforin	AMP	AG**R**G**K**QGG**K**V**R**A**K**A**K**T**R**SS**R**AGLQFPVG**R**VH**R**LL**RK**GNY	39	β-sheet
GAP43(p)IQ	CBD	AAT**K**IQA(*p*)SF**R**GHIT**RKK**L**K**GE**KK***DD*	25	β-sheet
IP3R1	CBD	**K**SHNIVQ**K**TALNW**R**LSA**R**NAA**R**	22	α-helix
IP3R2	CBD	EN**RK**LLGTVIQYGNVIQLLHL**K**S	23	β-sheet
RYR	CBD	**K**S**KK**AVWH**K**LLS**K**Q**RRR**AVVACF**R**MTPLYN	30	β-sheet
PMCA1	CBD	L**RR**GQILWF**R**GLN**R**IQTQI**R**VV**K**AF**R**SS	28	β-sheet
PMCA2	MBD	**KK**AV**K**VP**KK***E***K**SVLQG**K**LT**R**LAVQI	25	no
Control	—	*EE*HIY*DE*VAA*D*P	12	no

Peptides are classified here as antimicrobial peptide (AMP), calmodulin-binding domain (CBD), and membrane-binding domain (MBD), respectively. The group AMP involves membrane-active peptides reported to have antimicrobial activity like melittin, mastoparan, and CM15 as well as host-defense peptides like Dhvar4, and buforin. Calmodulin-binding domains are derived from the cytosolic target protein GAP43 (growth associated protein 43, peptide GAP43IQ), and membrane target proteins PMCA (plasma membrane calcium channel, peptide PMCA1), RYR (ryanodine receptor, peptide RYR), and IP3R (IP3 receptor, type 1, two peptides: IP3R1 and IP3R2). Peptide PMCA2 is the membrane-binding domain of the protein PMCA. For peptide GAP43IQ, there is a phospho-pair (peptide GAP43pIQ) bearing a phosphoryl group on the Ser where the protein GAP43 is phosphorylated *in vivo*. For more details see text and Methods section. Positively charged, negatively charged, and tryptophan residues in the primary sequence are bold, italic, and underlined, respectively. Secondary structure gained upon interaction with LPA is also indicated.

To detect structural changes caused by LPA, CD spectra recorded in the presence of the lipid were analysed (Fig. 1, Table 1, and Table S1 in Supporting Information). Definite changes were observed, which were consistent with a disorder-to-order transition within the peptides upon interaction with LPA. However, the nature of the structural arrangement showed clear differences among the peptides. Two peptides (mastoparan, and IP3R1) showed an increased α-helical content, whereas most of the peptides were found to gain β-sheet conformation. For melittin, a mixed conformation with dominant helix and some sheet contributions was detected. Interestingly, melittin adopted the most folded state induced by LPA as indicated by the dramatic decrease in the disordered fraction. The β-conformation in the presence of the lipid is very similar to that of the IP3R2 alone, which indicated that LPA did not influence the folded state of this peptide. Similarly, no remarkable structural change was detected for the control peptide that retained its disordered nature in the presence of LPA. For this case, the lack of the interaction can be explained with the ionic repulsion between the negatively charged residues and the phosphate moiety of the lipid head-group. In contrast, GAP43pIQ also bearing a phosphoryl-group in the middle, and two Asp residues at the C-terminus could seemingly adopt the same conformation upon addition of the lipid as its non-phosphorylated pair did.

It should be noted that aggregation upon addition of LPA could be detected for several complexes. This is most obvious in the spectrum of buforin where the negative peak at around 230 nm characteristic for the β-conformation almost disappeared while the positive part became bumpy. Similarly, pronounced intensity loss was experienced for IP3R1 with a helix-like spectrum. In line with the spectral changes, an increase in the sample absorbance was observed possibly due to the presence of higher oligomers formed with LPA.

### Tryptophan fluorescence indicates more apolar peptide microenvironment upon binding to LPA

**Figure 2:**
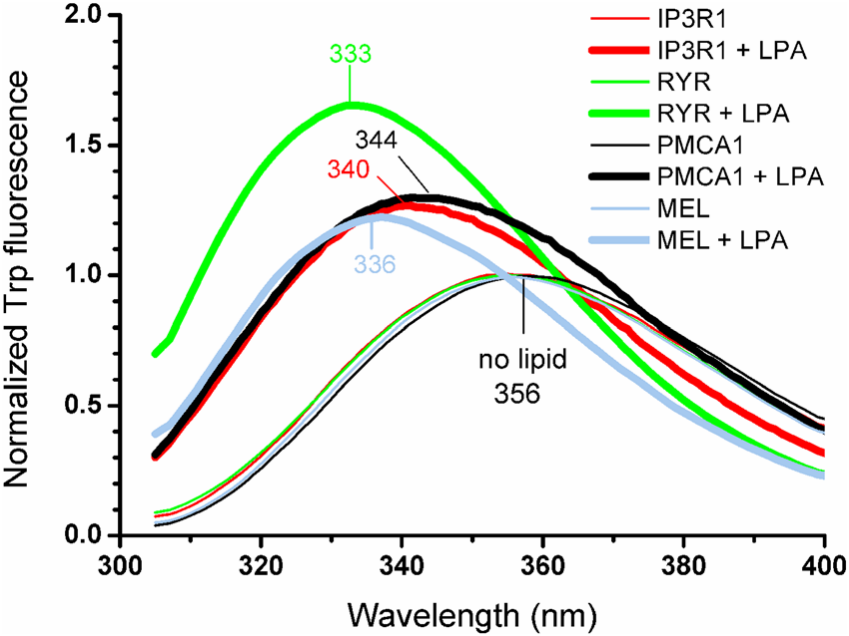
Lipid-peptide interactions studied by tryptophan (Trp) fluorescence. Spectra were taken at peptide concentration of 3 μM with and without 100 μM LPA in high-salt buffer, and normalized pairwise to the maximal intensity (I_max_) measured in the absence of the lipid. Note that each peptide alone showed emission maximum at 356 nm resulting in overlapping spectra which blue-shifted upon LPA addition as labelled in the figure, and listed in Table 2. Emission intensity variations at 1, 3, 6, and 10 μM are compared in Fig. S2.

To further characterize the LPA-peptide interactions, we utilized fluorescence spectroscopy. Tryptophan emission is very sensitive to the polarity of the environment of the fluorophore, which could provide a sensible tool to detect binding of a partner. We investigated four peptides containing tryptophan residue (melittin, IP3R1, RYR, and PMCA1). Without LPA, peptides at concentrations of up to 10 μM showed emission spectra with a maximum of 356 nm (Fig. 2, thin lines) suggesting a water-accessible tryptophan of presumably monomeric peptides^21^. Upon addition of the lipid at a concentration of 100 µM, a significant elevation in the emission intensity accompanied by a blue shift of the emission maximum was observed (Fig. 2, thick lines), which is consistent with the relocation of the tryptophan into a more hydrophobic environment upon binding to the lipid. The shift showed no correlation with the conformation adopted (Table 2). An emission maximum at ~340 nm measured for IP3R1 and PMCA1 can be interpreted as partially buried tryptophan residues, or a mixture of fully exposed and fully buried species, whereas the highest lipid phase participation is suggested for RYR exhibiting the largest blue shift^22^. Nevertheless, the shift observed for each peptide was clearly independent of the lipid-to-peptide ratio, and the maximal intensities followed a linear function in the concentration range used, *i*.*e*. 1–10 μM and 100 μM for the peptide and the lipid, respectively (Fig. S2). This indicated that peptides bound subsequently to the lipid could probably adopt the same conformation upon complex formation.

**Table 2:** Peptide tryptophan fluorescence in the presence and absence of LPA.

Peptide	λ_max_ (nm)		Relative I_max_	Induced secondary structure
	no LPA	100 μM LPA	(LPA/no LPA)	
PMCA1	356	344	1.34	β-sheet
IP3R1	356	340	1.29	α-helix
MEL	356	336	1.22	α-helix (+β-sheet)
RYR	356	333	1.69	β-sheet

Data refer to the spectra shown in Fig. 2. Secondary structures are according to the CD spectroscopy-based evalution.

### Comparison with other lysophospholipids and detergents

**Figure 3:**
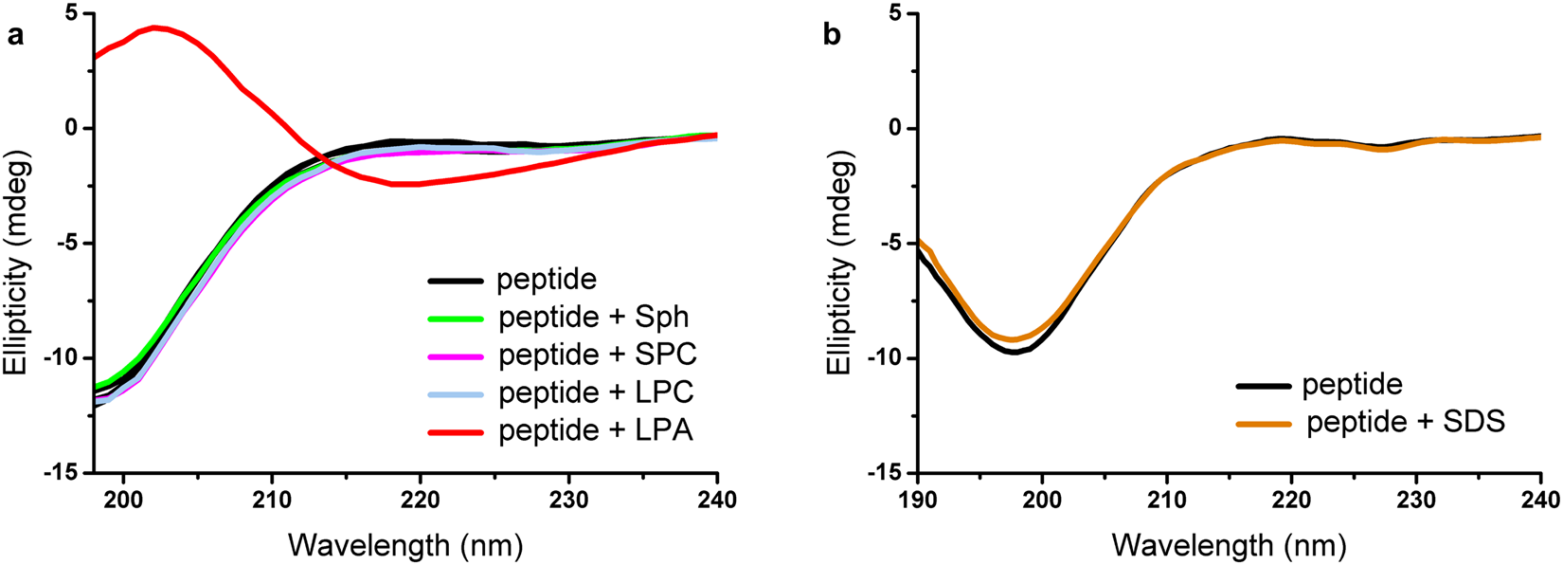
LPA selectivity among the investigated peptide-lipid interactions. CD spectra for GAP43pIQ (36 µM) were measured in the presence and absence of lysophospholipids (lysophosphatidylcholine, LPC, sphingosylphosphorylcholine, SPC, and sphingosine, Sph) at 100 µM in high-salt buffer (**a**), and SDS at 100 μM in low-salt buffer (**b**), respectively. Only LPA affected peptide conformation.

To determine lipid selectivity of the lipid-peptide interactions, spectra of peptide GAP43pIQ in the presence of LPA and structurally related lysophospholipids bearing similar acyl chains but different head-groups with various charge distributions were compared utilizing CD spectroscopy (for structure of the lipid species see Fig. S1 in Supporting Information). No change in the spectra upon addition of lysophosphatidylcholine, sphingosylphosphorylcholine, and sphingosine was observed indicating peptide binding selectively to LPA at low ionic strength. Specificity obtained this way could be interpreted in terms of electrostatically driven initiation of the interaction. The same effect was observed under high-salt conditions (Fig. 3), which underlines the importance of the nature of the lipid head-group in the interaction, and also verifies the reliability of the binding event at physiological salt concentrations.

The effect was also probed in the presence of sodium dodecyl sulfate (SDS), the anionic detergent well-known for its ability to alter peptide conformation. No spectral change could be detected at 100 µM SDS (Fig. 3) but the negative result could easily be explained with a different lipid state at this concentration. Both SDS and LPA form micelles in aqueous solutions, the different behaviour could be connected to their different association properties in line with alterations in the head-group, and the shorter acyl chain (C12) of SDS compared to that of LPA (C18). Micelle formation is characterized by the CMC (critical micelle concentration) which value was reported to be ~25–50 μM for LPA, and in the low millimolar range for SDS, respectively^23,24^. Considering that the CMC for an ionic lipid can significantly vary with the ionic strength, we have also measured the CMC in the assay buffers used. We have exploited the ability of pyrene to incorporate easily into hydrophobic patches formed by lipid aggregates, which is accompanied by the change in its fluorescence emission. Similar CMC values of ~20–40 μM at 25 °C were determined for LPA in high-salt and low-salt buffer (Fig. S3 in Supporting Information), respectively, which is also in good agreement with the value determined previously by microcalorimetry in phosphate-salt buffer at 37 °C^24^. Using the same pyrene-based method, a CMC of ~2 mM was obtained for SDS in low-salt buffer, similarly to as reported before^23^, which is two orders of magnitude higher than that for LPA. Surfactants were also reported to form non-micellar associates, clusters even at concentrations well below their CMC^15,25^. Determination of CMC could therefore interfere with detection of these sub-micellar lipid clusters as published for some neutral surfactants, the clusters of which were shown to accomodate pyrene^25^. Nevertheless, CMC values for anionic surfactants determined by pyrene fluorescence agreed well with those obtained by surface tension methods^15,25^, which argues for measuring real micelle formation concentrations for SDS and LPA in our case.

### LPA incorporated to liposomes can induce structural changes similarly to LPA micelles

LPA associates formed in aqueous solutions are micelles that might well represent the high curvature of an LPA patch formed locally upon LPA production under *in vivo* conditions. In general, probing vesicles containing the lipid of interest seems to be an attracting way to test the effect of a membrane component lipid under more biological conditions. However, when investigating non-permanent membrane-constituting signalling lipids like LPA, reconstitution of the lipid in a biologically relevant, locally enriched form resembling the distribution in natural membranes can be challenging, even when phase separation was supposed for sterol-containing multicomponent vesicles^26^. Nevertheless, the ability of LPA to insert into liposomes as model membranes was validated^27^. Although the current study primarily focuses on interaction with LPA associates, to address differences in interactions depending on LPA environment, similarly as comparing LPA with detergents like SDS, we have performed experiments with liposomes incorporating LPA.

**Figure 4:**
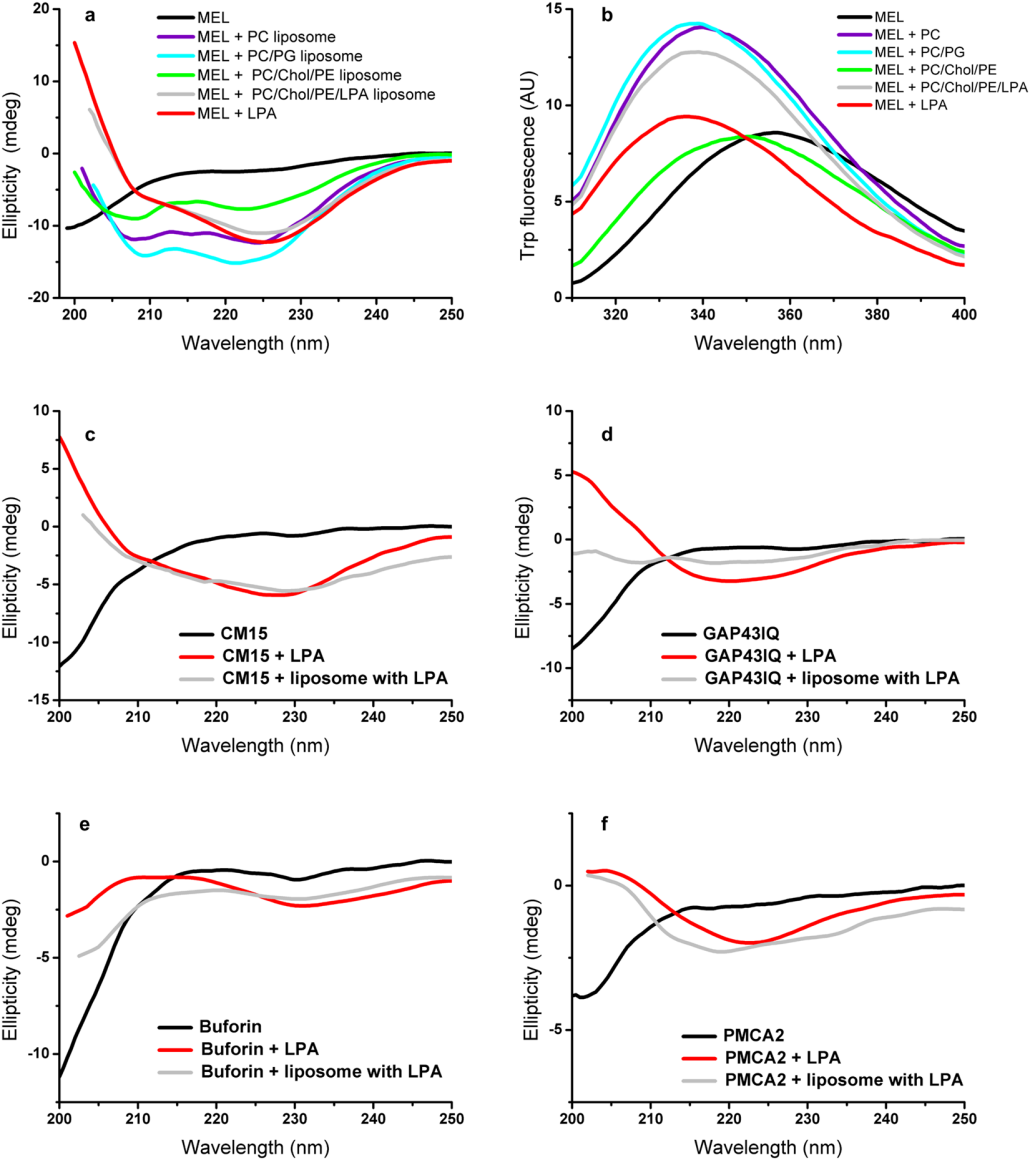
Peptide interaction with liposomes containing LPA. CD spectra (**a**), and fluorescence emission spectra (**b**) for melittin (30 and 2 µM, respectively) in the presence of various liposomes. (**c**–**f**) CD spectra of peptides CM15 (36 µM), GAP43IQ (36 µM), buforin (36 µM), and PMCA2 (17 µM) in the presence of LPA-containing liposomes. All spectra were recorded in high-salt buffer. Spectra taken with LPA micelles (100 μM lipid) are also shown for comparison. For molar composition of the liposomes see Methods section. Nominal lipid concentrations for the liposomes were as follows: (**a)** PC, and PC/PG 1.3 mM, PC/Chol/PE and PC/Chol/PE/LPA 2 mM, (**b)** 100 μM for all liposomes. (**c**–**f**) PC/Chol/PE/LPA 1 mM.

First we investigated the well-studied melittin in the presence of various liposomes differing in composition. Melittin is known for its capability to bind to both neutral and anionic membranes as well as detergents resulting in gain in ordered secondary structures^28,29^. The helical conformation induced by the zwitterionic phosphatidylcholine (PC), and the negatively charged phosphatidylglycerol-containing (PC/PG) liposomes differed from the helical one adopted in the presence of LPA-containing liposomes and micelles manifested in alterations in the relative intensities of the helix bands at 208 and 222 nm in their CD spectra (Fig. 4a). The peptide structure adopted when bound to the cholesterol and phosphatidylethanolamine-containing phosphatidylcholine-based (PC/Chol/PE) liposome with no LPA resembled that measured with pure phosphatidylcholine (Fig. 4a). Using LPA-containing liposomes (PC/Chol/PE/LPA), the same helical transformation was observed for melittin as with LPA micelles (Fig. 4a). As for further comparison, the CD spectrum recorded with SDS micelles was similar to the phosphatidylcholine bilayer-bound helix conformation^29^.

According to the fluorescence emission values in the 336–339 nm range (Fig. 4b), melittin tryptophan is located in an apolar environment in all lipid-bound cases except the PC/Chol/PE liposome, the latter showing weaker binding in CD assays (Fig. 4a) as well. The most buried tryptophan with the most blue-shifted emission maximum could be assigned for melittin interacting with micellar LPA. Note that the different fluorescence intensities due to possible quenching in different local environment indicate some positional perturbations despite forming a similar secondary structure as observed by CD spectroscopy.

To further explore the effect of LPA incorporated into model membranes, several peptides studied here were selected and their CD spectra were recorded using LPA-containing PC/Chol/PE liposomes. When comparing these results with those obtained with LPA micelles, similar spectral features were detected with peptides CM15, buforin, and PMCA2 (Fig. 4c,e,f) suggesting similar binding events with micellar and bilayer-incorporated LPA associates. The nearly identical spectra for CM15 (Fig. 4c) indicated a mixed helical and sheet-like conformation with higher β-sheet contribution in both LPA environments. The minimum in the 220–230 nm range was present for the complex with buforin and PMCA2 (Fig. 4e–f), in agreement with increased β-sheet content upon interaction with LPA-containing liposomes. Following the trend, the calmodulin-binding domain GAP43IQ also showed elevated ordered structure content upon interaction with liposomes incorporating LPA according to the increased intensities of the shoulder at 220–230 nm and reduced intensities at around 200 nm, respectively (Fig. 4d). It should be noted that peptide interaction with LPA-containing vesicles could be accompanied with aggregation, which assembly process occured during the binding event with LPA micelles as well.

Taken together, our results with liposomes suggested that membrane-incorporated LPA may exert similar structural effects as the micellar lipid form arguing the micellar state as a good model for LPA interaction studies. These results also indicated that the presence of LPA could induce subtle conformational changes even on peptides with low lipid specificity like melittin being active with various kinds of lipid compositions and morphologies (Fig. 4a). While very similar secondary structures are formed for both LPA micelles and LPA-containing liposomes, the fluorescence studies demonstrated that relative positioning of membrane-active peptides may change depending on the lipid morphology (Fig. 4b). Nevertheless, membrane-linked LPA may have only minor conformational effect on peptides or protein segments with a more sensitive or specific sequence, for which more strict conditions are needed to fold, only fulfilled by the high curvature of micellar or membrane-born LPA.

### The affinity and stoichiometry of the peptide-LPA interactions

To determine binding affinity of the lipid-peptide interactions, titration experiments were performed utilizing CD and fluorescence spectroscopy as well as isothermal titration calorimetry (ITC). For these measurements, the β-sheet forming GAP43IQ peptide was selected. Since GAP43IQ lacks tryptophan residues, fluorescence-based experiments were conducted with the β-sheet forming RYR, and the α-helix forming IP3R1 peptides. Results obtained with LPA were compared with those with SDS.

**Figure 5:**
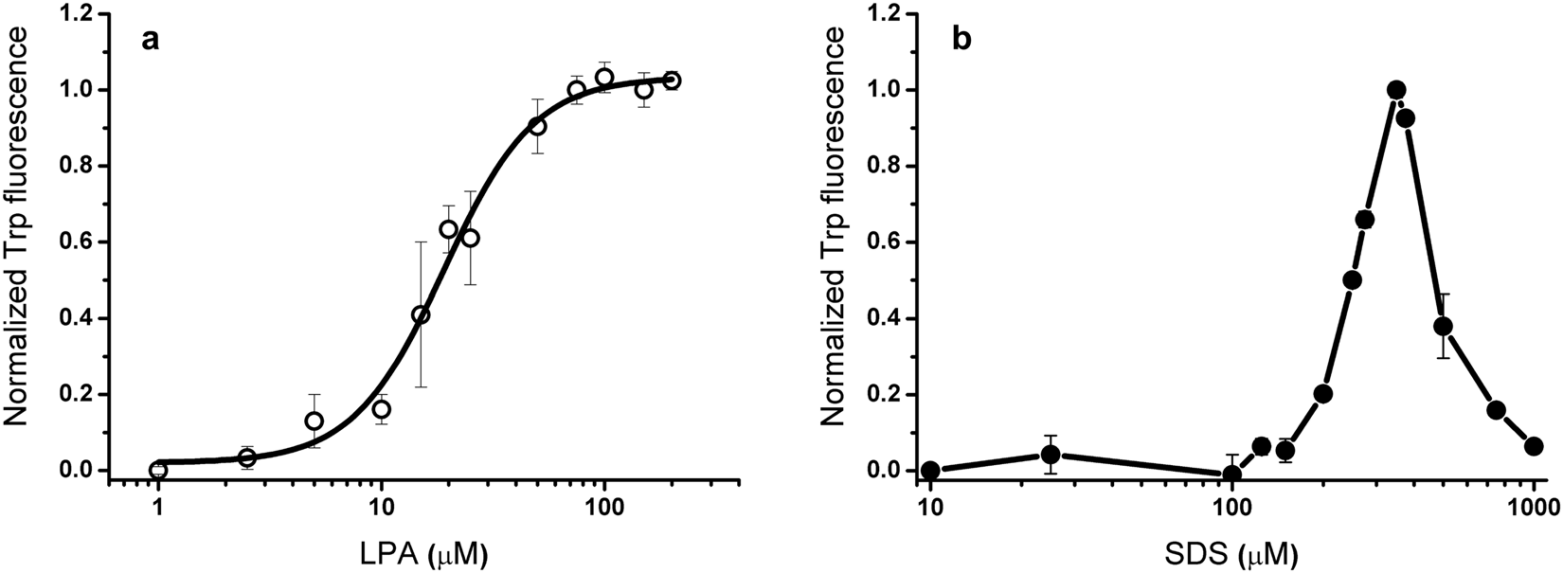
Titration of IP3R1 with LPA (**a**) and SDS (**b**) exploiting tryptophan (Trp) fluorescence. Titration with LPA was performed in high-salt buffer while that with SDS in low-salt buffer, also used for CD spectroscopic experiments. Data were fitted to the Hill-equation, and yielded an apparent *K*_d_ of 19.0 ± 1.3 μM and a Hill-coefficient of 2.1 ± 0.36 for the IP3R1-LPA interaction. Values are mean ± SEM (n = 3).

Using tryptophan fluorescence, titration of IP3R1 with LPA in high-salt buffer resulted in a simple sigmoid dose-response curve with an apparent dissociation constant (*K*_d_) of 19 μM (Fig. 5a). This value is very close to the CMC determined under the same condition. A similar value of 20 μM was obtained for the RYR peptide. Considering that IP3R1 gained α-helix whereas RYR had increased β–sheet structure, this observation indicated that peptide folding driven by the lipid is not dependent of the particular conformation to be formed. Titration result also suggested that LPA was able to bind to the peptides in an associated form, which is, based on the CMC data (Fig. S3 in Supplementary Information), the micellar state. In contrast, binding of the peptide to SDS resulted in a bell-shaped lipid-dependence curve with a maximum of ~350 μM when plotting maximal fluorescence intensities against the lipid concentration (Fig. 5b), indicating a more complex binding mechanism. Nevertheless, the concentration range at which the binding event was detected is much below the CMC, indicating peptides probably contacting lipid clusters in this case. Alternatively, formation of shared micelles consisting of peptides and lipids resulting in an apparent lowering the CMC in the presence of peptides may also be a likely scenario^15^.

**Figure 6:**
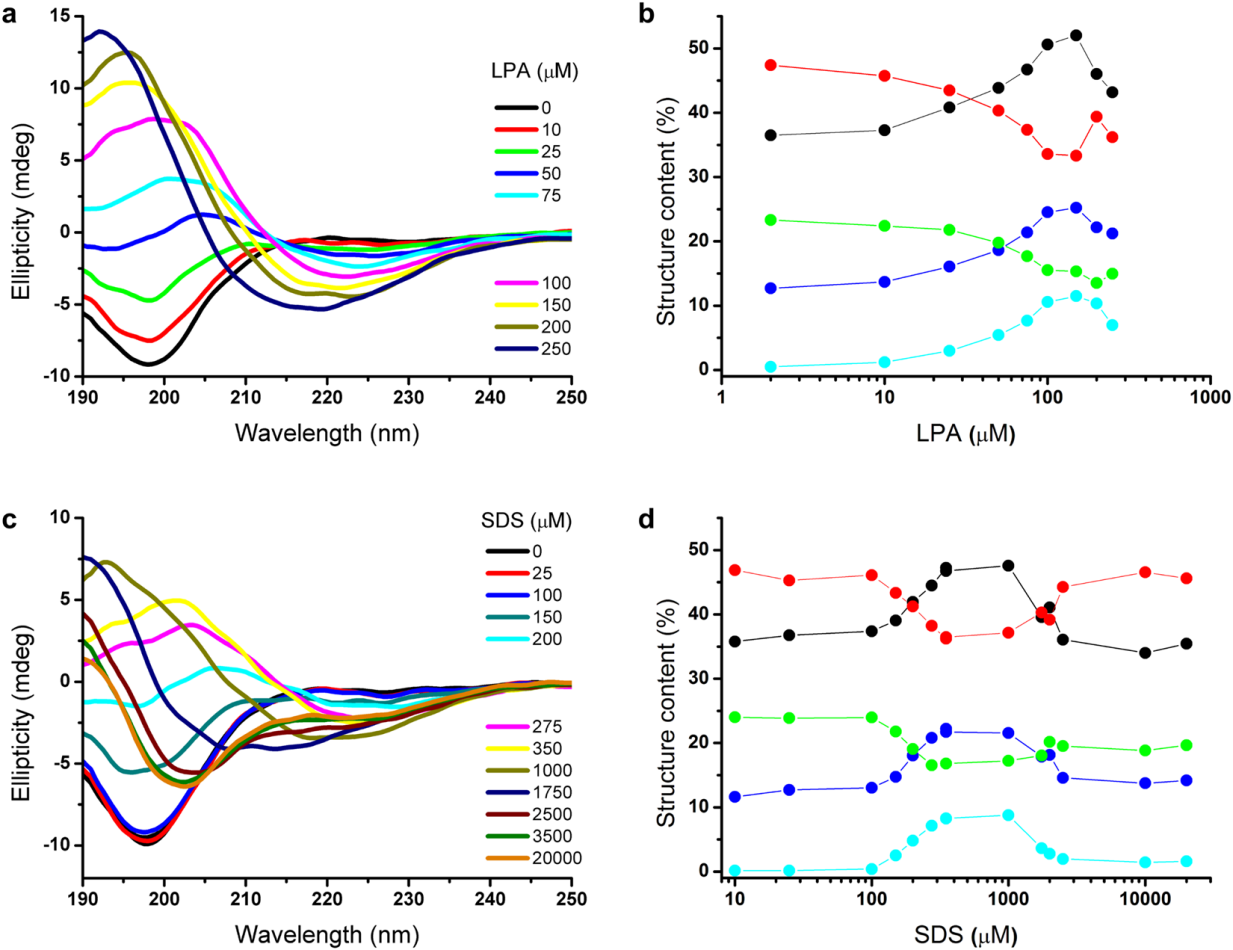
Structural changes of peptide GAP43IQ induced by LPA and SDS traced by CD spectroscopy. (**a**–**c**). Spectra of the peptide recorded in the absence and in the presence of the lipids. (**b**–**d**) Lipid concentration-dependent changes in peptide conformation highlighting elements with pronounced alterations upon interaction. Secondary structure elements are according to the classification of the analysis method used considering three types of antiparallel β-sheet with different twists (cyan, blue and green). The content of all the individual β-forms, the total estimated β-conformation (black), and the disordered fraction (red) changed in the same lipid concentration range. Note that structural changes in the presence of SDS and LPA follow similar trends but take place at different concentrations, for LPA at CMC and for SDS much below the CMC.

To address lipid-dependent structural changes in the peptide conformation, GAP43IQ was titrated with LPA while differences were followed by CD spectroscopy (Fig. 6). It is clearly seen that with raising the LPA concentration, and thus lipid-to-peptide ratios, the β-sheet content increased at the expense of the unstructured content. The effect occured at lipid concentrations at which micelles form, and saturated at 100–200 μM LPA. Similar spectral changes could be observed for the SDS titration, but at a much higher concentration, in the 350 μM–1 mM range. Above the observed plateau, an opposite effect with elevation of the helical content dominated at about the CMC, so that the peptide structure in the presence of excess SDS micelles (above 2 mM) resembled rather the conformation adopted in the absence of SDS.

**Figure 7:**
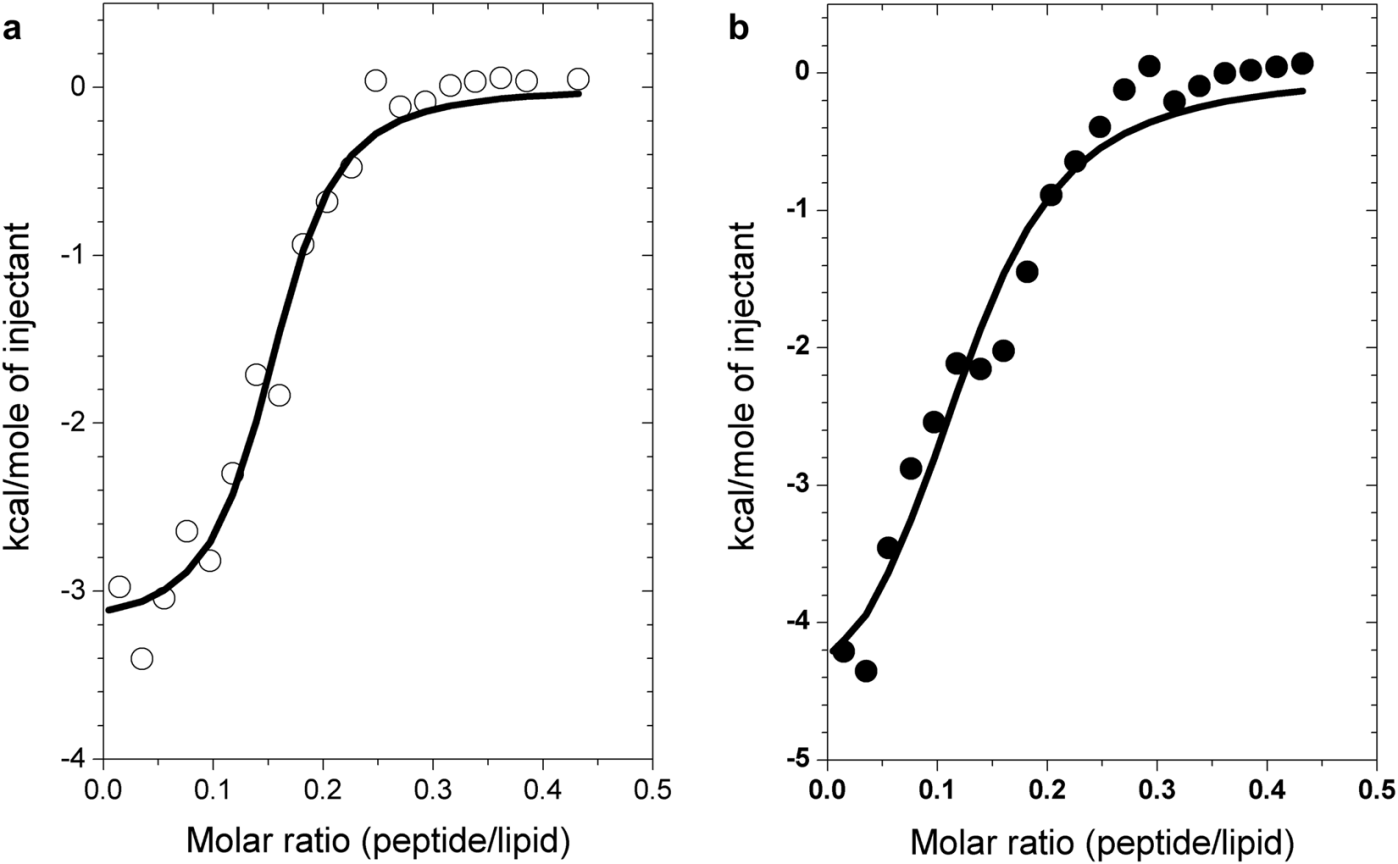
Calorimetric traces for the LPA-peptide interaction. LPA (100 µM) was titrated with aliquots of the peptide GAP43IQ (200 μM) under high-salt (**a**) or low-salt (**b**) conditions. Points were fitted to the *one set of sites* model (solid lines), and parameters evaluated are listed in Table 3.

Lipid titrations utilizing fluorescence and CD spectroscopy yielded an apparent binding constant of ~20–40 µM for the LPA-peptide interaction. This apparent K_d_ is resulted from the convolution of binding and the lipid aggregation processes. Moreover, our results suggested binding of several peptides to associated LPA. To determine intrinsic affinity constants, stoichiometry, as well as thermodynamic parameters dictated by the driving forces of the interaction, ITC measurements were carried out. LPA at a concentration above the CMC, *i*. *e*. a mixture of LPA monomers and micelles, was titrated with the peptide GAP43IQ under both low-salt and high-salt conditions (Fig. 7). With this setup, parameters for the binding of one peptide to one apparent lipid binding site could be deduced. Based on the fluorescence spectroscopic findings above, where subsequent peptide binding resulted in identical peptide tryptophan environment, data were fitted to the *one set of sites* model assuming several identical binding sites (Table 3). Considering that a more complex binding scenario including several binding sites of different affinities may be plausible as indicated by some deviation of the fitted curve at higher peptide-to-lipid ratios, we have also employed the *two sets of sites* model, resulting in similar estimates as above. The fitting process yielded a peptide-to-LPA ratio of 1:7-1:8 in the complex in both low-salt and high-salt assay buffers. As micelles typically consist of ~60–100 monomers^24^, this strongly suggests that several peptides could bind to a single LPA micelle. Similar binding strength in the high-nanomolar and low-micromolar range under high-salt and low-salt conditions, respectively, was estimated (Table 3). Considering the thermodynamic parameters obtained, the negative enthalpies could be indicative of favourable electrostatic and hydrogen bonding contributions. On the other hand, the negative −*TΔS* entropic term can be interpreted as favourable conformational changes and/or hydrophobic interaction during binding.

**Table 3:** Thermodynamic parameters of the LPA-GAP43IQ interaction obtained from the ITC measurements depicted in Fig. 7.

Condition	n (peptide:lipid)	K_d_ (μM)	*ΔH* (kcal/mol)	*−TΔS* (kcal/mol)
high-salt	~1:7	0.62 ± 0.21	−3.2 ± 0.15	−5.2
low-salt	~1:8	2.4 ± 0.85	−5.0 ± 0.54	−2.7

### LPA-peptide complex formation under crowding conditions

Structural aspects of the peptide-LPA interaction were also investigated by means of infrared spectroscopy using the ATR-FTIR technique. We focused on the amide I band representing the hydrogen-bonding pattern of the peptide backbone, thereby sensitively monitoring the secondary structure of peptides^30^. Spectra for both the hydrated form in solution, *i*. *e*. non-dried, and the surface-dried samples were analysed in order to reveal possible effects on the complex formation due to changes in the hydration shell and the elevated concentration. Conditions in the dried film might mimic a crowding milieu with a high local concentration and reduced set of surrounding water molecules.

**Figure 8:**
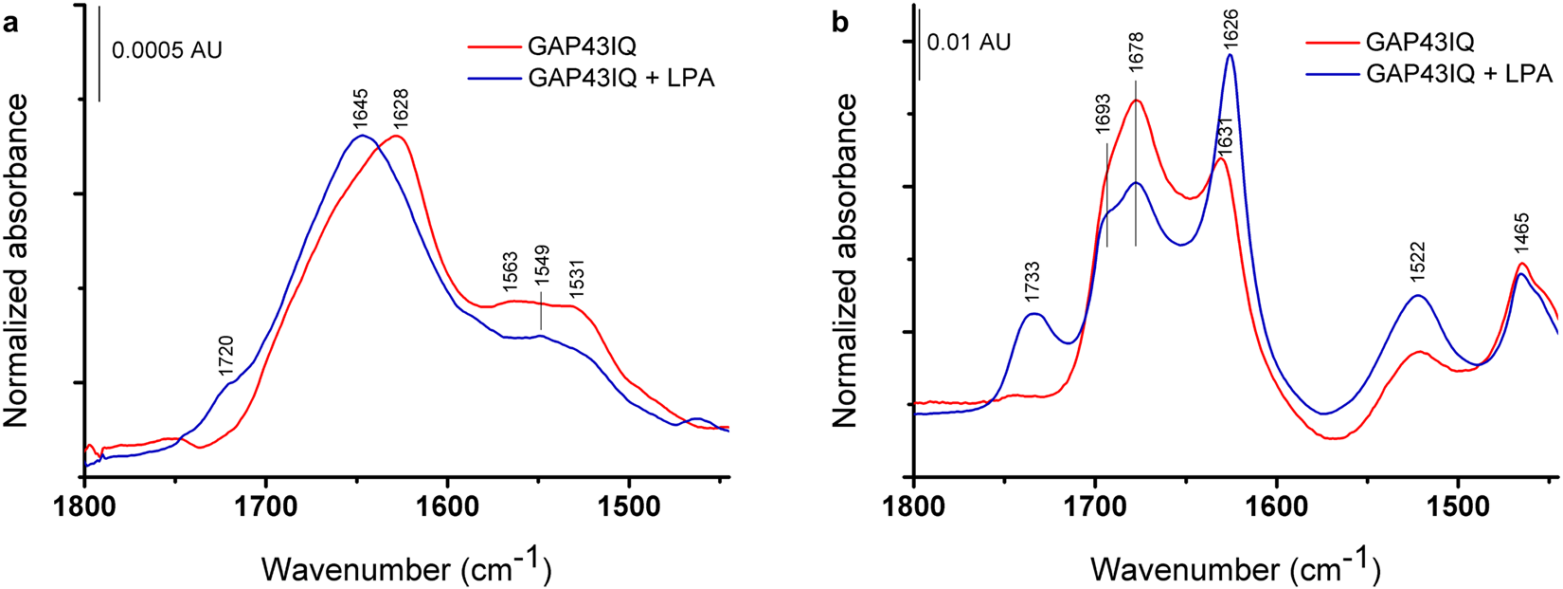
The effect of LPA on the peptide structure studied with ATR-FTIR. Complex formation was initiated by mixing the components with a peptide-to-lipid ratio of ~1:5 using concentrations of 200 μM and 1 mM for the peptide and the lipid, respectively. Spectra were collected for the mixture immediately after mounted (solution, **a**) as well as for the surface-dried sample (**b**), respectively.

The case of GAP43IQ is displayed (Fig. 8) as a representative example of a peptide adopting β–sheet conformation in the presence of LPA based on CD results. Compared with a dominant disordered structure suggested by CD measurements, GAP43IQ without the lipid in the hydrated form (Fig. 8a) showed signs of some self-assembly as indicated by the presence of an amide I band component at ~1620 cm^−1^, possibly due to the higher concentrations applied in the IR experiments. This is in agreement with the reported ability of amphipathic peptides to form oligomers in aqueous solutions at higher concentrations as exampled by the well-studied melittin tetramer^21^. The addition of LPA reduced the intensity of this low-wavenumber band component in expense of elevation of the band centre at ~1650 cm^−1^ (Fig. 8a), which can be explained in terms of separating the peptide monomers by the added lipids. This behaviour is in agreement with multimeric peptide binding in the micellar interior suggested by fluorescence and ITC experiments. In contrast, a more pronounced peptide-peptide interaction upon complex formation was seen for the dried samples (Fig. 8b) highlighting the role of the surrounding water molecules available.

### Structural aspects of the LPA-peptide interactions

Based on CD spectroscopic results, we demonstrated that the lipid mediator LPA could drive secondary structure formation within unstructured peptides sharing a basic amphiphatic nature. According to the general view, calmodulin-binding domains bind as a regular α-helix to calmodulin, as reported for a couple of these motifs derived from calmodulin-dependent protein targets^31^. Similarly, for most of the AMPs used here, the helical conformation appeared to be the membrane-active form. In line with these, insect venom-derived peptides melittin and mastoparan, as well as calmodulin-binding motif IPR3P1 folded to a more helix-rich secondary structure in the presence of LPA. In contrast, most of the investigated peptides involving human host-defense peptides and calmodulin-binding segments of target proteins showed markedly increased β-sheet content upon addition of LPA.

**Figure 9:**
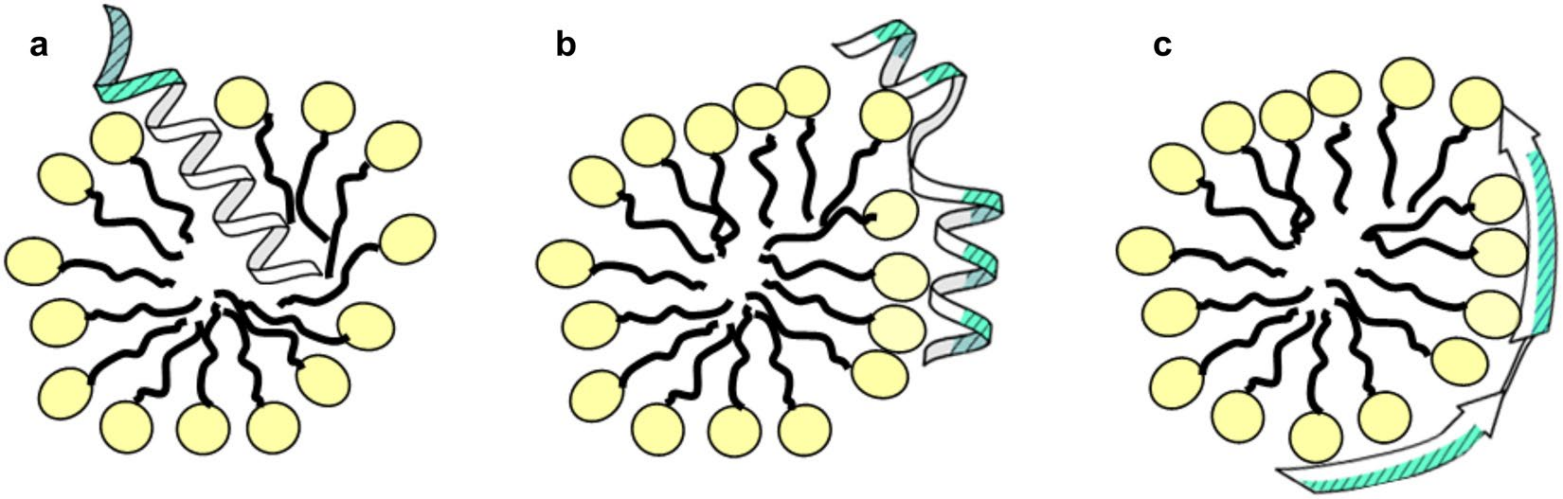
Schematic model showing three possible arrangements for the investigated set of peptides upon interaction with LPA micelles. (**a**) A peptide in inserted helix conformation, (**b**) a peptide in helical conformation associating with the micelle, (**c**) a peptide in extended, sheet-like conformation. Note that in cases **b** and **c**, peptide hydrophobic side chains are most likely inserted into the more lipophilic area, beyond the headgroup region of the micelles. Location of hydrophilic residues is highlighted by dashed cyan fill on the corresponding side of the formed secondary structure. For the complete set of peptides and their side chain distribution upon forming helical or extended sheet-like conformation, see Fig. S4 in Supplementary Information.

In order to understand the potential reasons leading to molecular level structural differences between these peptides, we have investigated the peptide sequences focusing on the hydrophobic and hydrophilic, charged side chains, as well as how these are oriented in a helical, or sheet-rich conformation (Fig. S4 in Supporting Information). Based on this comparison, as well as taking into account all the measured data presented herein, for the investigated set of peptide sequences we outline three schematic modes of interactions (Fig. 9). Previous results on AMPs and membrane protein sequences clearly indicated that longer apolar sequences prefer helical conformation and membrane insertion, as the helix formation covers the polar peptide groups by forming inner hydrogen bonds shielded by the apolar side chains, which prefer to leave the aqueous phase in presence of lipid bilayers^32^. It is indicative that for melittin and mastoparan, there is a rather long sequential region where almost exclusively hydrophobic amino acids are present, while the charged residues are also concentrated to one of the terminal regions. Considering also, that the tryptophan of melittin showed a marked change in fluorescence, these suggest that melittin is likely to be inserted in a preferentially more helical form into the LPA micelles with its hydrophobic region deeply inside the micelle core (Fig. 9a). Mastoparan probably occupies the headgroup region of the micelles where its apolar side chains are in the acyl chain region. In case of the third helical sequence, peptide IP3R1, the most probably reason is similar to that of mastoparan: here the charged cationic residues are separated by shorter apolar sequences in such a manner, that, when forming a helix, all basic residues are located on the same side of the peptide (Fig. 9b). This would suggest that in this case the helical structure could reside on the surface of the micelles with hydrophilic side facing water, similarly as AMPs executing carpet mechanism^2^. In contrast, the remaining set of investigated peptides show increased levels of β-sheet content. Most of these sequences contain polar and apolar side chains intermixed, where a more extended conformation can help arranging residues with opposing polarity to separate sides (Fig. 9c). Note that for the latter case it is unclear whether β-sheet content is also accompanied with increased oligomerization or not, a structural aspect beyond our current focus.

Furthermore, besides revealing the structural determinants of the LPA-peptide interaction, our findings suggested an electrostatically initiated complex formation followed by contacts with hydrophobic and hydrogen-bonding contributions. However, our results also pointed out that the charge distribution along the peptide could be more important in the proper contact between the peptide and the lipid than simple electrostatic effects. IR-based results underlined the importance of hydration and revealed a dynamic complex formation resulting in alterations of the complex in more crowding conditions.

The fluorescence studies raised the possibility of oligomeric peptide binding to LPA, which assumes interaction with lipid associates. This idea was supported by ITC titration findings. Moreover, our IR data are in accordance with a binding process where folding is coupled to oligomer formation as also suggested by CD titration results.

Compared with SDS, structural variations caused by LPA were found similar suggesting a detergent-like effect, however, the fact that LPA was shown to be effective at about the CMC while SDS much below the CMC, argued for different mechanisms (Fig. 6). Similarly, the same difference in the effective concentration was reported for the two compounds in promoting fibril-formation of the globular blood protein β_2_-microglubulin^24^. In addition, aggregation of the intracellular intrinsically disordered protein GAP43 (growth associated protein 43) was observed only at about 0.01% SDS^33^, the same concentration range at which we experienced peptide structural changes (~350 μM, Figs 5 and 6). These results indicate a different intrinsic property of SDS and LPA associates to interact with peptides and proteins.

### Implication of the peptide-LPA interaction in AMP action

LPA is present in various biological fluids at a physiological concentration in the low micromolar range^34^, however, its blood level can be increased several-fold under certain pathological conditions^16,34,35^. It should be emphasized that its local concentration could reach levels used in the study, especially, if taking into account that the reported values represent spatio-temporal averages. Considering its extracellular accumulation, LPA might interact with host-defense or antimicrobial peptides circulating in blood, therefore, could be able to modulate their action.

An emerging set of evidence points to multiple mechanisms of action of AMPs, targeting not only membrane lipid components but also intracellular molecules, well illustrated by mastoparan. Mastoparans are a family of peptides from wasp venom composed of 14 amino acids varying in primary sequence. Their mechanism of action involves, followed by internalization into host cells, direct activation of GTP-binding regulatory proteins, stimulation of nucleotide exchange on G proteins^36^ located at the cytosolic surface of the plasmalemma. Only mastoparan variants forming helices in the presence of usual lipid bilayers have shown regulatory activity on G proteins, and, based on CD data, the helix conformation reported in the presence of phosphatidylcholine liposomes^36,37^ was very similar to the mastoparan helix adopted upon interaction with LPA (Fig. 1). In line with this, production and accumulation of LPA on the inner leaflet of the plasma-membrane might interfere with the presentation of multiple cationic structures required for G protein regulation.

### Implication of the peptide-LPA interaction in modulation of protein function

Following the considerations above, possible role of LPA in modulating function of biomolecules is probable. The idea that LPA can elicit effects on intracellular proteins was supported by the reported interaction with the actin-regulator villin^38^ or with the suggested intracellular LPA receptor PPARγ^39^. Recently, we identified the SH3 domain of the signalling protein Caskin as the membrane-binding part of the protein showing high affinity for associated LPA^27^. In line with this, membrane-born signalling lipids like LPA might control the function of calmodulin-dependent membrane-bound proteins, and mediate protein sequestering to the membrane. Our results with protein binding motifs might also point out to regulation of protein function by LPA as follows.

Protein interactions are often modelled using peptide binding segments, and the approach is widely used in studying calmodulin function *in vitro*, as reviewed^31,40^. This concept can lead to reliable results only when the peptide binding motif separated from the macromolecule interacts in the same way as when it was connected to the parent protein. On the other hand, proteins often utilize a peptide segment to attach to the membrane, exploiting the amphipathic property of a membrane-binding domain. In both cases, the disordered peptide part folds to a well-defined secondary structure.

The functional promiscuity due to structural similarity is highlighted by the integral membrane protein PMCA (plasma membrane calcium channel) possessing both calmodulin-binding and membrane-binding segments. The membrane-binding peptide fragment was reported to bind to calmodulin even when with a moderate affinity, however, the calmodulin-binding segment showed higher affinity for model membranes than the dedicated membrane-binding part of the pump^41^. The corresponding peptides used in our study (PMCA1 and PMCA2, respectively) behaved similarly in LPA-binding assays showing similar structural arrangement in the presence of LPA (Fig. 1).

Another example for this diverse function is the calmodulin-binding domain of the cytosolic, intrinsically disordered protein (IDP) GAP43 (growth associated protein 43) suggested to regulate calmodulin function (reviewed in ref.^42^). Membrane attachment of their complex is presumably achieved *via* a segment partially overlapping with the CaM-binding domain (IQ motif, peptide GAP43IQ in our study), thereby, interaction of GAP43 with membranes competes with the calmodulin-binding ability. The peptide GAP43IQ was widely used to model binding properties of the whole protein^43,44^. Since IDPs hardly crystallize, structural information on the interaction was gained based on the x-ray structure of the calmodulin-GAP43IQ complex^45^ showing the IQ domain in an α-helical conformation. Partial folding of the motif to α-helix was also observed in the presence of liposomes incorporating various lipids bearing negatively charged headgroups^46^. Here we report that associated LPA is an effective structure inducer of the peptide part, too. Moreover, we identified LPA as a selective lysophospholipid binding partner of the parent protein GAP43 as well, detailed characterization of which interaction will be published separately.

### Specific interactions with endogenous compounds resulting in rare conformations

Our results comparing several lipids above have demonstrated that LPA exerts specific interaction with the investigated peptides, which does not depend merely on its physicochemical properties, as structurally related lysophospholipids have not induced significant structural changes as LPA. Note, that similar selectivity was observed recently in our group when exploring interactions of disordered cationic AMP sequences with small anionic organic molecules, and endogenous bile pigments of hemin and its metabolites^47,48^. These studies represent several groups of compounds with similar molecular setup, nevertheless, in all groups we found both compounds which induced drastic conformational changes on AMPs and compounds showing nearly no interaction with the investigated peptides. This phenomenon on several examples strongly suggests that there is a more complex selection mechanism, other than the mere mix of counter charges and apolar regions, occuring at the molecular level. Most likely, the sequential distribution of charged and hydrophobic or aromatic residues on the AMPs is also a crucial prerequisite to facilitate the formation of the observed complexes resulting in peptide conformational changes.

Further on, it is also interesting to note, that among the compounds actively changing conformation of the investigated AMPs and disordered protein regions, several has endogenous origin. While these are generally present in low concentrations *in vivo*, there are several scenarios, usually related to external stress conditions, where elevated concentration of *e*.*g*. hemin, biliverdin, or LPA occur. For instance, increased hemin level clearly takes place during hemolytic activity of certain bacterial species, whereas LPA concentration is increased during cancer. Although exact identification of these interactions and potential modulatory mechanisms is very challenging, and thus beyond our current focus, it worths mentioning that the increasing amount of examples on strong AMP-endogenous molecule interactions may hint towards a more complex regulatory process present during these events.

Finally, for melittin, and additional AMPs, these interactions have resulted in rather uncommon, rare folded peptide conformations, which are not characteristic for these sets of AMPs. Along this line, Takahashi *et al*. has also demonstrated that giant vesicles can induce a strong β-sheet formation for melittin^49^. These indicate that in presence of endogenous compounds *in vivo*, the most efficient toxic conformation of host-defense peptides may be altered, or even be very different from those observed and presented during *in vitro* experiments. This, currently plausible scenario, may be related to sudden increase of antimicrobial efficiency similar to a recently demonstrated synthetic compound with optical control instead^50^. Nonetheless, it is clearly an exciting direction to be investigated, as these molecular level structural changes may also provide indications on how host organisms retained efficiency of their antimicrobial peptides also against resistant bacterial strains.

## Conclusion

Here we demonstrate the ability of LPA to effectively drive folding of disordered cationic amphipathic peptides. For most of the investigated sequences, induced folding resulted in secondary structures that are not commonly observed for these compounds. The comparison of LPA with structurally related lipids and detergents has referred to a specific interaction of the peptides with LPA where the crucial parameter is connected to its association capacity already at low concentrations. Our findings, in line with the emerging data pointing to multiple mechanisms of actions of AMPs involving peptide assembly and lipid clustering, suggest role for the lipid mediator LPA in modulating the action of biomolecules like peptides and proteins. These indicate that the biological function of AMPs in *in vivo* conditions might be related to a more complex mechanism where non-standard conformations are induced either for complementary functions or for an effective toxic action.

## Methods

### Assay conditions

Two assay conditions were used thoroughly in the study. HEPES-based high-salt buffer (10 mM HEPES, 100 mM KCl, pH 7.2) mimicking physiological conditions was used commonly. In cases when K^+^ or Cl^−^ ions should be avoided, low-salt buffer (25 mM Na-phosphate, pH 7.0) was used.

### Peptide solutions

Peptides were synthesized by the providers as follows: melittin, GAP43(p)IQ, and the control peptide by EZBiolab (Carmel, IN, USA), IP3R1, IP3R2, RYR, and MAS by Bio-Science Trading Ltd (Hungary). The PMCA-derived peptides and CM15, dhvar4, buforin were a kind gift of Dr. Ágnes Enyedi (Budapest, Hungary) and Dr. Szilvia Bősze (Budapest, Hungary), respectively. Peptides used are i) calmodulin-binding domains of membrane target proteins PMCA (plasma membrane calcium channel, PMCA1^41^), RYR (ryanodine receptor^51^), IP3R (IP3 receptor, type 1, two segments: peptide 1 and 2^52,53^), ii) calmodulin-binding IQ domain of the cytosolic target protein GAP43 (growth associated protein 43, neuromodulin, peptide GAP43IQ^45^ and its phosphorylated pair GAP43pIQ^54^), iii) membrane-binding domain of PMCA (PMCA2^41^), and iv) membrane-active, host-defense peptides classified as antimicrobial peptides (AMPs) like melittin, mastoparan, CM15, dhvar4, and buforin. The sequence and some properties of the peptides used in this study are listed in Table 1.

Peptide solutions were prepared either in ultrapure water (MilliQ) or in high-salt buffer.

### Lipid solutions

Lipids for the binding assays were purchased from Avanti Polar Lipids (Alabastar, Alabama, USA): lysophosphatidic acid, 18:1 LPA (1-oleoyl-2-hydroxy-*sn*-glycero-3-phosphate, sodium salt, 857130), lysophosphatidylcholine, 18:1 LPC (1-oleoyl-2-hydroxy-*sn*-glycero-3-phosphocholine, 845875), sphingosine, Sph (D-erythro-sphingosine, 860490), sphingosylphosphorylcholine, SPC (860600), phosphatidylcholine, PC (1,2-dioleoyl-*sn*-glycero-3-phosphocholine, 850375), phosphatidylglycerol, PG (1,2-dioleoyl-sn-glycero-3-phospho-(1′-rac-glycerol), sodium salt, 840475), phosphatidylethanolamine, PE (1,2-dioleoyl-*sn*-glycero-3-phosphoethanolamine, 850725), cholesterol, Chol (700000). 10–50 mM stock solutions were prepared in methanol or chloroform.

Before each experiment, lipid solution was prepared by drying the necessary lipids into glass vials, and resuspending in the assay buffer by vigorous sonication and vortexing. For preparing SDS (sodium dodecyl sulphate) solutions, SDS powder (Sigma, L3771) was dissolved in assay buffer avoiding K^+^ ions that form precipitate with SDS. For some experiments, liposomes were also used. Pure PC, and PC/PG (80/20 n/n%) liposomes (10 mg/ml, 12.7 mM total lipid) were prepared by drying the lipids in glass vials, dissolving them in the assay buffer by alternated vortexing and sonication, followed by repeated freeze-thaw cycles and extruding through a polycarbonate filter with a pore diameter of 200 nm. LPA-containing (PC/Chol/PE/LPA 40/25/15/20 n/n%) and control (PC/Chol/PE 60/25/15 n/n%) liposomes (2 mM total lipid) were prepared by drying the lipids in glass vials, dissolving them in the assay buffer by alternated vortexing and sonication, followed by centrifugation at 100,000 g for 30 minutes, and reconstitution of the pellet in the high-salt assay buffer. The centrifugation step is needed to separate liposomes from LPA micelles that might be formed upon hydration of the dry lipid film. Due to the separation step, concentration values for these liposomes are nominal.

### Circular dichroism spectroscopy

Spectra were collected using a Jasco-720 spectropolarimeter at 25 °C in low-salt or high-salt buffer. High-salt buffer mimics salt concentrations present in *in vivo* environments. At least three spectra were recorded in the far-UV region (190–250 nm in low-salt buffer, and 200–250 nm in high-salt buffer, respectively) at a speed of 50 nm/min with a bandwidth of 1 nm using a thermostattable, cylindrical, quartz cuvette of 1 mm path-length. Spectra were corrected by subtracting the blank (lipid containing buffer), and smoothed. For lipid selectivity experiments (using LPA, LPC, Sph, SPC), peptide and lipid concentrations were 20–50 μM and 100 μM, respectively. For lipid titration measurements, spectra for the peptide GAP43IQ in the presence of various concentrations of LPA (up to 250 μM) and SDS (up to 20 mM) were taken. For liposome binding assays, 1–2 mM lipid and 26–36 μM peptide concentration was used. Note that for samples containing liposomes, signals are not reliable below 202–205 nm due to light scattering and thus these wavelength regions were not considered in the corresponding figures. Spectrum analysis was performed using the single or multiple spectra analysis option at the BeStSel online analysis tool^20^ (http://bestsel.elte.hu).

### Fluorescence spectroscopy

Spectra were collected using a Jobin Yvon Fluoromax-3 spectrofluorimeter at 25 °C in low-salt buffer or high-salt buffer. Spectra were recorded three times, averaged, and corrected by subtracting the blank (lipid containing buffer). Testing the peptide-lipid binding with peptides containing tryptophan, the fluorophore was excited at 295 nm, and emission was monitored from 305 to 400 nm. Peptide and lipid concentrations were 1, 3, 6, 10 μM and 100 μM, respectively. Probing the affinity of the peptide-lipid interactions, 2 μM peptide was titrated with LPA (0–200 μM). The maximum intensities were read and fitted to a sigmoid function using the Origin software (MicroCal, MA, USA). Results are mean ± standard error of mean, SEM (n = 3). Measuring liposome binding, 2 μM melittin was used in the presence of various liposomes at 100 μM.

The CMC (critical micelle concentration) of LPA and SDS was measured fluorometrically by monitoring the incorporation of pyrene. 1 μM pyrene was excited in the presence of various amounts of lipid at 332 nm, emission was monitored from 345 to 500 nm^25^. The ratio of the intensities at 373 nm (I_1_) and 384 nm (I_3_) were plotted against the lipid concentration (0–250 μM for LPA, and 0–20 mM for SDS, respectively).

### Attenuated total reflectance Fourier-transform infrared (ATR-FTIR) spectrometry

ATR-FTIR spectra were acquired using a Varian 2000 FTIR Scimitar Series spectrometer fitted with a *Golden Gate* single reflection diamond ATR accessory (Specac Ltd, UK). 5 μl of sample was mounted on the diamond ATR crystal. The measurements were done at room temperature immediately after mounting (*i*. *e*. in the solution) as well as after drying the sample by slow evaporation of the buffer solvent under ambient conditions. 64 scans were co-added at a nominal resolution of 2 cm^−1^. Prior to spectral evaluation, the buffer background was subtracted, and all spectra were smoothened with a Savitzky-Golay convolution function. The GRAMS/32 software package (Galactic Inc, USA) was used for all spectral manipulations.

### Isothermal titration calorimetry

Thermodynamic parameters for the interaction of peptide GAP43IQ and LPA were examined using a VP-ITC_200_ instrument (MicroCal, MA, USA). Measurements were performed at 25 °C in low-salt and high-salt buffer. Aliquots of GAP43IQ (200 μM) were injected into the ITC cell containing 100 μM LPA in the same buffer. Titration curves were analysed using the Origin for ITC software provided by MicroCal.

## Electronic supplementary material

Supplementary Information

## Data Availability

Data generated or analysed during this study are included in this article and its Supplementary Information file, and are available from the corresponding authors upon request.
