# Supplementary material for: The lipid mediator lysophosphatidic acid induces folding of disordered peptides with basic amphipathic character into rare conformations
Source: Sci Rep. 2018 Sep 28;8:14499. doi: 10.1038/s41598-018-32786-4 (PMC6162328; doi:10.1038/s41598-018-32786-4)

The lipid mediator lysophosphatidic acid induces folding of disordered peptides with basic amphipathic character into rare conformations

Tünde Juhász*, Judith Mihály, Gergely Kohut, Csaba Németh, Károly Liliom, Tamás Beke-Somfai*

Supplementary Information

Figure S1. Structure of the lipids used in the binding assays. Lipids and detergents used thoroughly in the study are as follows: 18:1 LPA (1-oleoyl-2-hydroxy-*sn*-glycero-3-phosphate, lysophosphatidic acid), 18:1 LPC (1-oleoyl-2-hydroxy-*sn*-glycero-3-phosphocholine, lysophosphatidylcholine), SPC (sphingosylphosphorylcholine), Sph (D-erythro-sphingosine, sphingosine), and SDS (sodium dodecyl sulphate). Drawings were taken from the Avanti Polar lipids website (https:\\avantilipids.com).

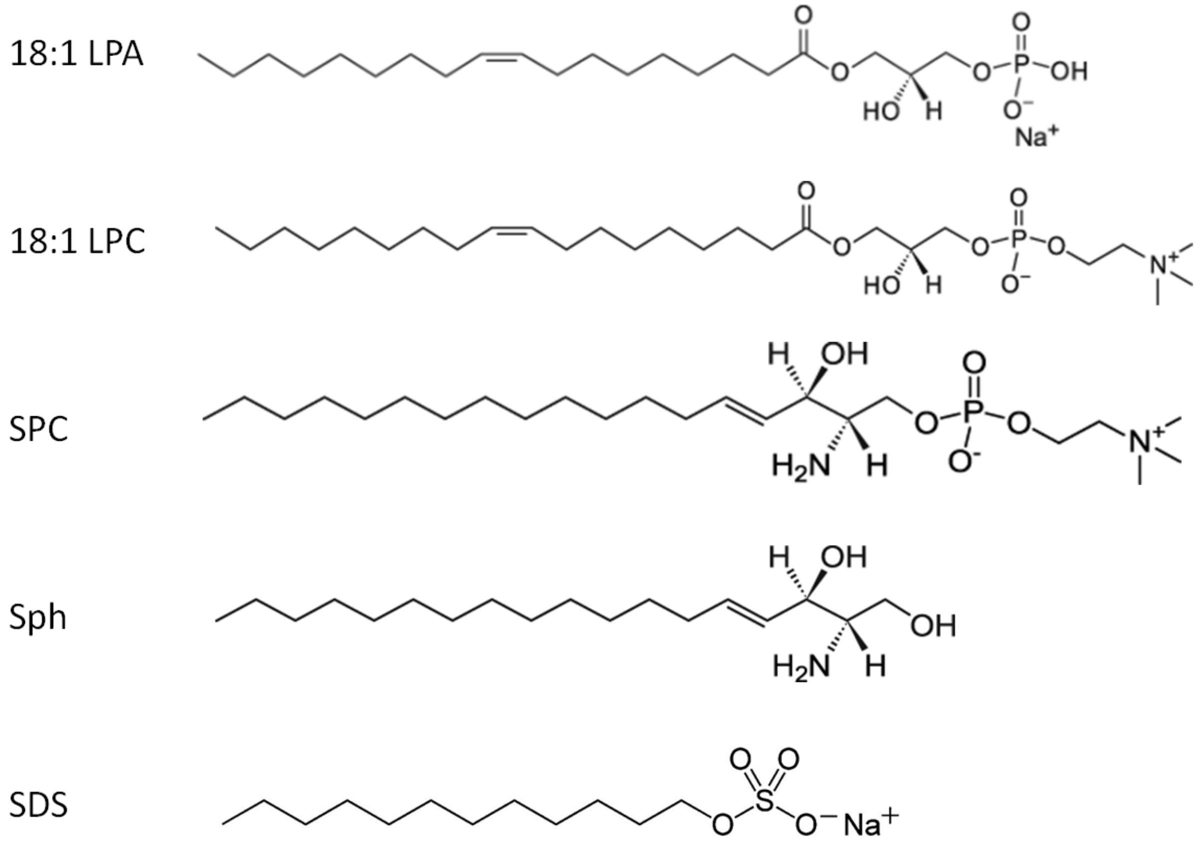

Table S1. Changes in the secondary structure of the peptides upon addition of LPA. Secondary structure content was estimated using the BeStSel online tool, and refer to the spectra shown in Fig.1. Ordered structure gained is also indicated.

	**Helix**		**Antiparallel Beta**		**Parallel Beta**		**Turn**		**Others**		**With LPA**
**Peptide**	**no LPA**	**LPA**	**no LPA**	**LPA**	**no LPA**	**LPA**	**no LPA**	**LPA**	**no LPA**	**LPA**	**folding to**
MEL	16	57	23	8	3	7	12	15	47	13	α-helix
MAS	10	51	26	6	0	0	16	12	47	31	α-helix
IP3R1	0	12	37	33	1	0	16	13	46	42	α-helix
GAP43IQ	0	1	35	51	3	0	16	14	47	35	β-sheet
GAP43pIQ	0	0	34	51	2	0	16	15	48	34	β-sheet
CM15	11	0	21	40	0	12	16	21	51	27	β-sheet
PMCA1	0	1	34	45	4	9	15	14	47	30	β-sheet
PMCA2	2	0	29	46	6	0	16	18	47	36	β-sheet
RYR	3	5	32	30	1	12	16	15	48	38	β-sheet
Dhvar4	2	0	49	46	0	0	10	25	39	28	β-sheet
Buforin	0	0	33	46	6	0	14	14	47	38	β-sheet
IP3R2	0	0	56	58	0	0	17	18	27	24	no
Control	10	16	27	22	0	0	14	15	49	48	no

Figure S2. Fluorescence emission intensity changes of the peptides in the presence of LPA. Spectra were taken at peptide concentration of 1, 3, 6, and 10 μM with and without 100 μM LPA in high-salt buffer, and normalized pairwise at each peptide concentration to the maximal intensity (I_{max}) measured in the absence of the lipid. Each peptide alone showed emission maximum at 356 nm which blue-shifted upon LPA addition to the 333-344 nm region as labelled in Fig. 2, and listed in Table 2. Relative intensities at the emission maxima in the absence and the presence of LPA are shown at the peptide concentration indicated.

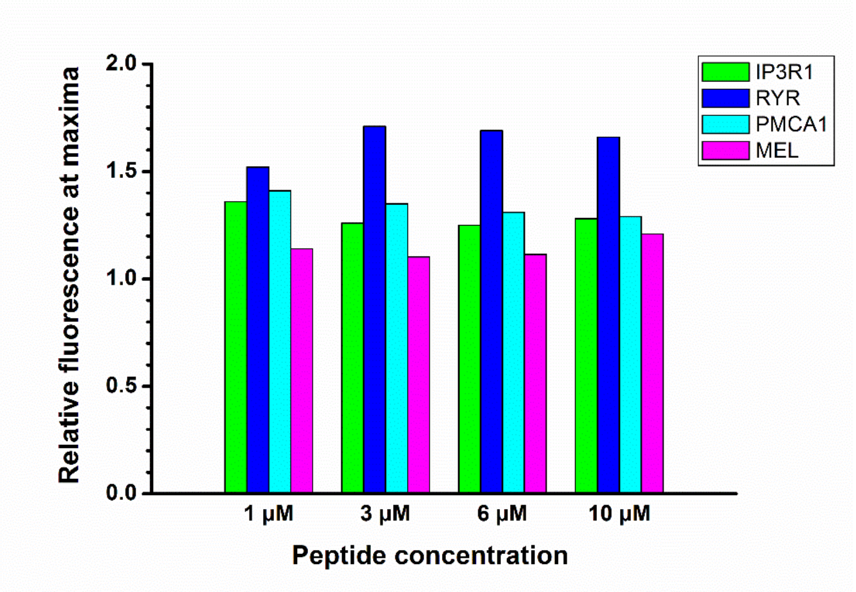

Figure S3. Determination of the CMC using pyrene fluorescence. CMC of LPA and SDS were measured in the low-salt and high-salt assay buffers used thoroughly in the study. The ratio of the emission intensities at 373 and 384 nm were plotted against the lipid concentration. Concentrations where $I_{373\ nm}/I_{384\ nm}$ tends to decline below the value of 1.4 characteristic for the free pyrene in aqueous solution were considered as CMC values (see the arrows).

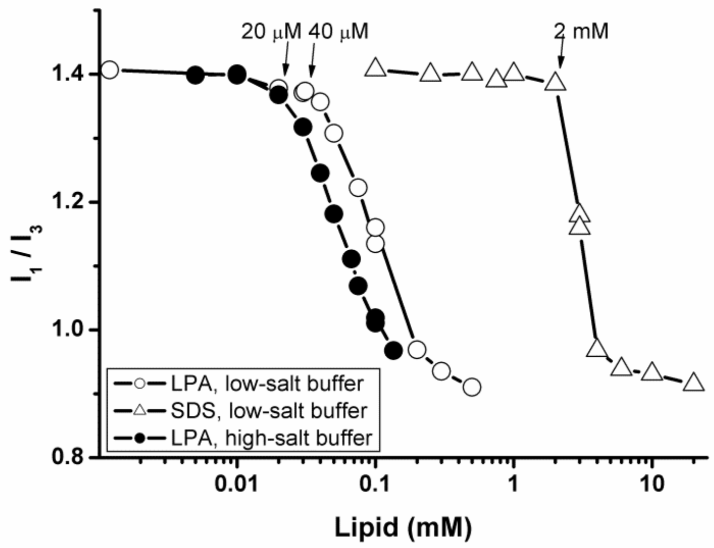

Figure S4. Side chain orientations of the peptides in β-strand (left) and α-helical (right) secondary structure. Conformers were generated based on the average torsion angles corresponding to either α-helix or β-sheet using the software VMD (University of Illinois)**.** Green color represents the non-polar sidechains, while blue shows the polar, basic and acidic ones.

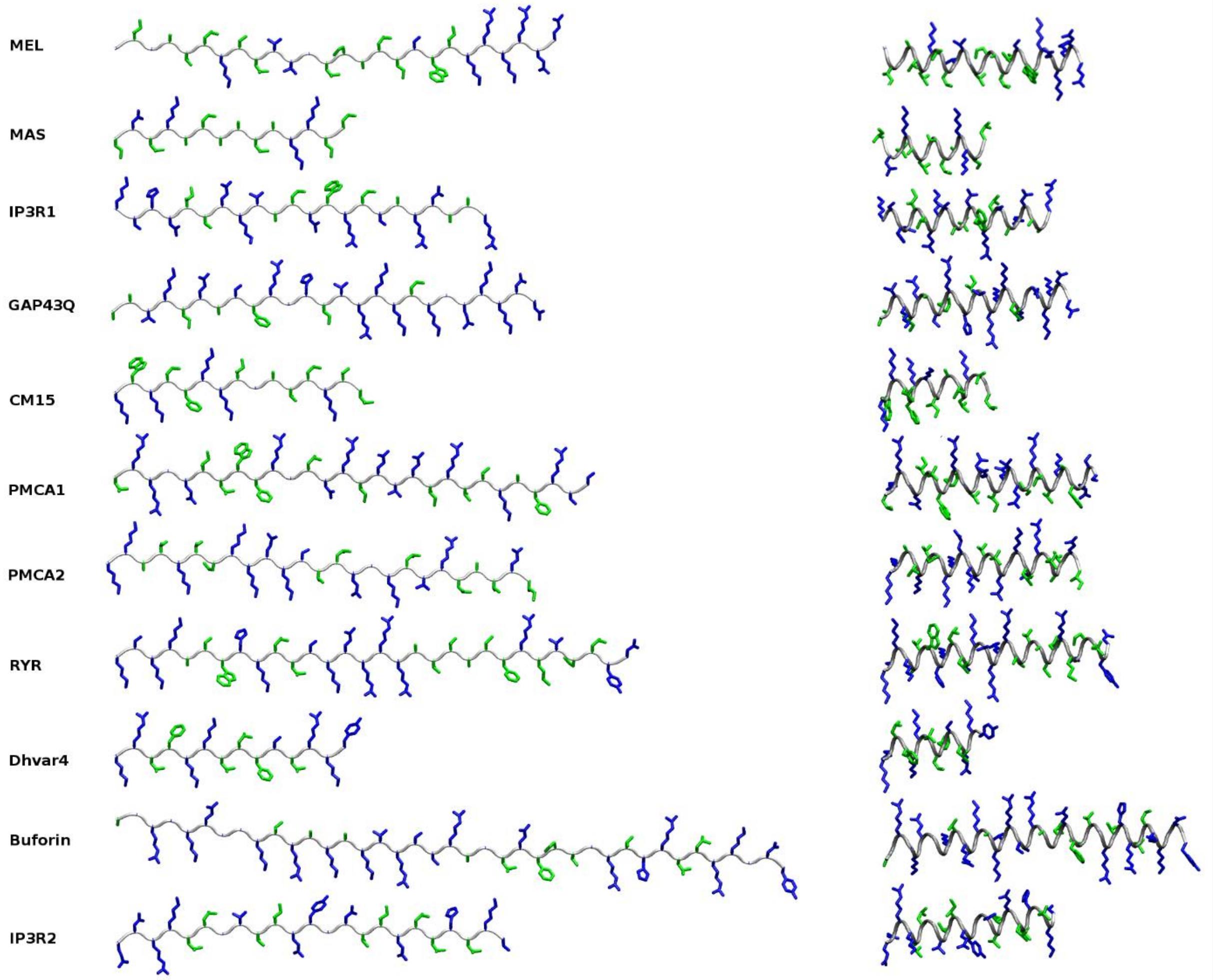

Supplement: Supplementary file 1 — Supplementary Information [file 41598_2018_32786_MOESM1_ESM.pdf]
